# Robot-guided stereotactic single‐stage evacuation of six intracranial abscesses: a rare case report with literature review

**DOI:** 10.1186/s41016-026-00436-8

**Published:** 2026-05-27

**Authors:** Yuhang Wei, Chen Zhu, Huaiyuan Wang, Junqi Wang, Pan Guo, Shaowu Ou, Jun Wang

**Affiliations:** Department of Neurosurgery, First Hospital of China Medical University, Shenyang, Liaoning 110001 China

**Keywords:** Brain abscess, Robot-assisted neurosurgery, Stereotactic aspiration, Minimally invasive surgery, Multifocal lesions

## Abstract

**Background:**

Multifocal, deep-seated brain abscesses are uncommon and often lead to rapid neurological decline because of mass effects, widespread edema, and the challenge of safely accessing multiple intracranial compartments. Traditional surgical approaches may require staged operations or extensive craniotomy, increasing procedural risk. Robot-assisted stereotactic aspiration offers a minimally invasive and highly precise alternative, yet its application in single-session, multitarget drainage remains rarely reported. This case describes a young patient with extensive bilateral brain abscesses that were successfully treated through one-stage, robot-guided multisite aspiration, highlighting the potential advantages of this technique in complex infectious neurosurgical emergencies.

**Case presentation:**

A 21-year-old woman presented with rapid onset of confusion, headache, and progressive neurological deficits. Magnetic resonance imaging revealed multiple deep abscesses involving the frontal, temporal, and occipital lobes, accompanied by severe cerebral edema and midline shift. After multidisciplinary evaluation, a single session of stereotactic aspiration was performed using a robot-assisted navigation system. Six abscess cavities were drained through four precisely planned trajectories during a single operation. Broad-spectrum antimicrobial therapy was initiated empirically and later tailored according to microbiological findings. A short-course of low-dose dexamethasone was added to reduce cerebral edema while minimizing the risk of impaired infection control. The patient demonstrated marked neurological improvement within several days and achieved full functional recovery within 1 month.

**Conclusions:**

This case illustrates that robot-assisted, minimally invasive stereotactic aspiration may represent a potentially safe and effective strategy for managing multifocal deep brain abscesses in selected patients, allowing accurate multitarget drainage in a single procedure. The rapid recovery observed in this patient supports the clinical value of integrating precise surgical intervention with optimized antimicrobial therapy and controlled edema management. This approach may offer a viable treatment pathway for similarly complex intracranial infections where conventional surgery is associated with significant risk.

## Background

Brain abscesses are serious neurological conditions characterized by localized intracranial infection, progressive mass effects, and potentially irreversible neurological impairment if not treated promptly. Multifocal and deep-seated abscesses present an even greater challenge because of their complex anatomy, heterogeneous etiologies, and high risk of deterioration. Conventional management typically involves antimicrobial therapy combined with surgical drainage, yet extensive or bilateral lesions often require staged procedures or wide surgical exposure, which may increase morbidity and delay infection control.

In recent years, minimally invasive stereotactic aspiration has become an important surgical option, offering precise access to abscess cavities while minimizing damage to healthy brain tissue. Robot-assisted stereotactic systems further improve accuracy, trajectory planning, and procedural safety, particularly for deep or multiple lesions. However, reports describing one-stage, multitarget aspiration for widespread multifocal brain abscesses remain scarce, and the clinical role of robotic guidance in such complex infection scenarios has not been fully defined.

The aim of this case report is to present a rare case of a young adult with rapidly progressive, multilobar brain abscesses successfully treated through single-session, robot-assisted aspiration of multiple deep lesions. This case highlights how precise minimally invasive drainage, integrated with optimized antimicrobial therapy and controlled edema management, can contribute to rapid neurological recovery. By discussing the clinical course, treatment strategy, and outcome, this report seeks to expand the current understanding of robot-guided interventions in complex intracranial infections and provide insight into their potential advantages in emergency neurosurgical practice.

## Case presentation

Intracranial abscess remains among the most challenging infectious diseases encountered in neurosurgical practice and is characterized by rapid progression, high morbidity, and potentially fatal complications such as herniation. Multifocal brain abscesses present particularly difficult management dilemmas because of their deep-seated location, multiplicity, and proximity to critical neural structures; therefore, they require rapid, precise intervention combined with coordinated medical therapy [1]. We report a case in which six intracranial abscesses were successfully treated in a single session using robot-guided stereotactic aspiration, combined with an integrated antimicrobial strategy and short-course low-dose corticosteroid for edema control. This case highlights the advantages of precision minimally invasive multitarget surgery together with an evidence-based perioperative pharmacologic strategy.

**Fig. 1:**
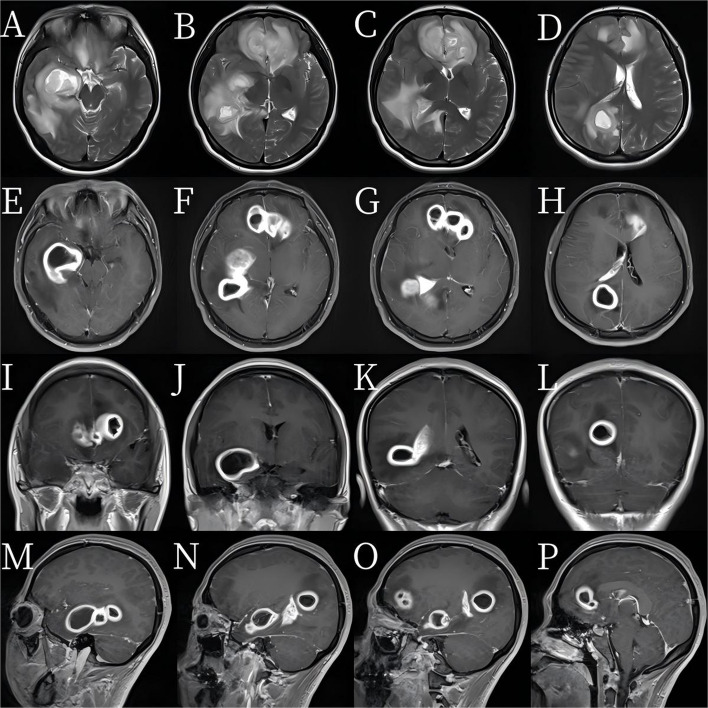
**A**–**D** Multiple mass-like hyperintense lesions on T2-weighted images are seen in the bilateral frontal lobes, right temporal lobe, and right occipital lobe. **E**–**P** Contrast-enhanced MR image showing prominent ring enhancement of the lesions, surrounded by extensive T2 hyperintense edema

A 21-year-old woman presented with a 7-day history of fever, headache, and vomiting. She had been empirically treated for *Mycoplasma pneumoniae* at a local hospital but developed progressive altered consciousness and was transferred to our neurosurgical unit. Upon admission to our neurosurgical center, she exhibited somnolence, with a Glasgow Coma Scale score of 10; neck stiffness; and positive meningeal signs, with a Karnofsky Performance Status (KPS) score of 70. Laboratory findings revealed leukocytosis and elevated C-reactive protein levels, indicating active infection. Magnetic resonance imaging revealed six ring-enhancing lesions in both frontal lobes and in the right temporal and occipital lobes. The largest lesion in the right temporal lobe measured 3.9 × 3.3 cm (Fig. 1). All the lesions displayed central necrosis, pronounced perifocal edema, and significant mass effects, with compression of the lateral ventricles and a leftward midline shift—features consistent with deep, multifocal brain abscesses.

**Fig. 2:**
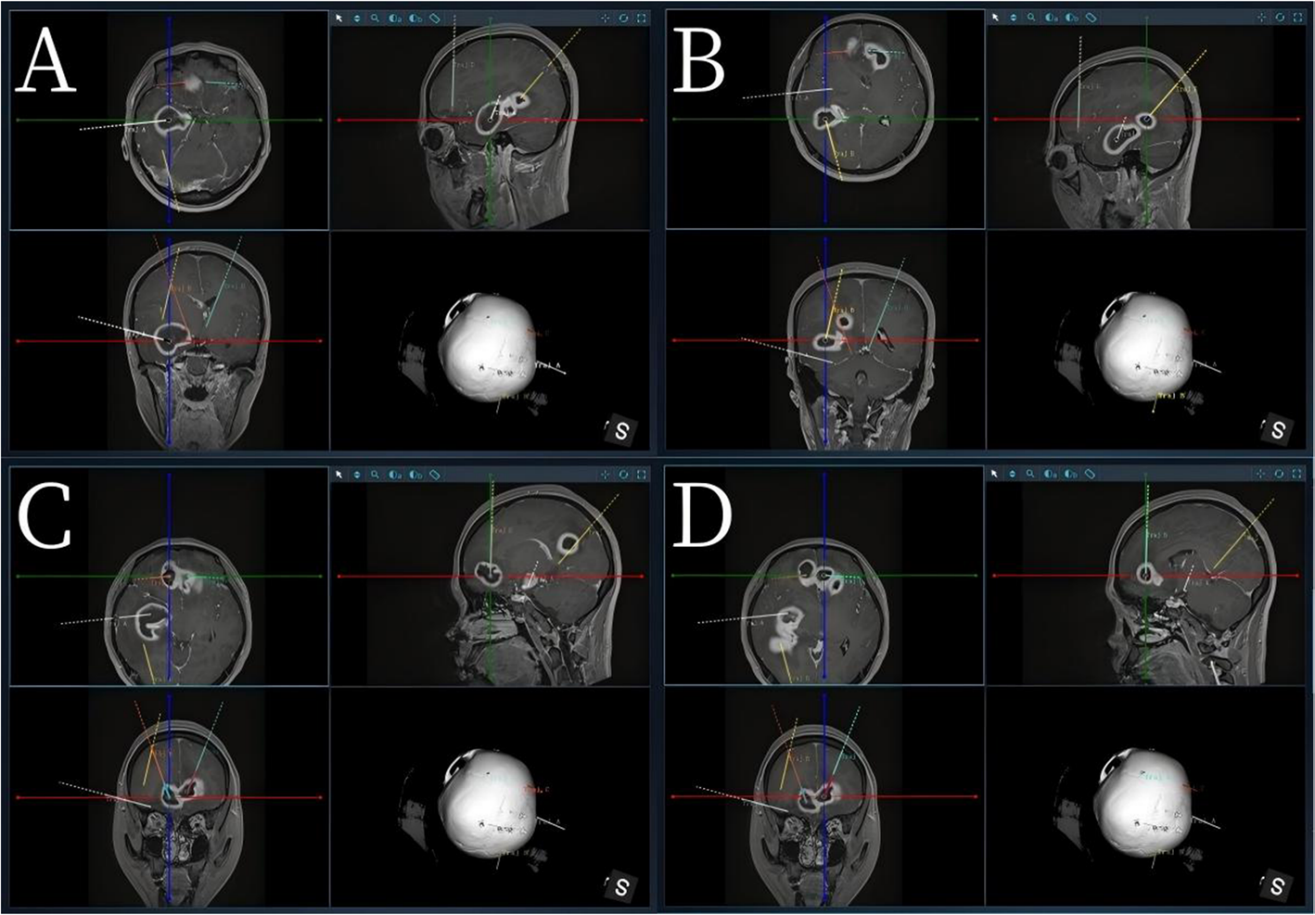
**A**–**D** Screenshot from Sinoplan software demonstrating trajectory planning based on the fusion of preoperative brain MR and CT images

Given the rapid neurological decline, radiographic mass effect, and short therapeutic window before impending herniation, conservative therapy alone was not appropriate. After multidisciplinary discussion (neurosurgery, infectious disease, neuroradiology, critical care), we selected emergent robot-assisted stereotactic aspiration as a minimally invasive, high-precision alternative to craniotomy or staged free-hand aspiration [2, 3]. Under cranial fixation, we performed an intraoperative CT that was fused with preoperative MR images using Sinovation Robot and SinoPlan planning software (Fig. 2).

Surgical planning: High-resolution 3D contrast-enhanced MRI was performed preoperatively, clearly delineating abscess cavities and adjacent critical structures, including cortical vessels, sulci, ventricles, and eloquent cortex. Intraoperative CT images were acquired and fused with MR images to create a precise 3D model. Using the SinoPlan robotic navigation system, abscess cavities and critical structures were segmented.

The lesions and surrounding anatomy were visualized in 3D. The trajectories were planned according to several principles: (1) shortest safe path to the abscess to minimize cortical penetration; (2) avoidance of critical structures (main functional areas, major vessels, sulci, ventricles); and (3) parallel alignment with major white matter tracts to reduce functional disruption.

Entry points were planned into the posterior or inferior third of the abscess to allow optimal drainage.

For lesions aligned along similar axes, sequential access through a single cortical entry was used to minimize cortical trauma. On the basis of the aforementioned design principles, we designed four puncture paths to access the six brain abscesses. Among them, two of the trajectories followed a “one puncture, two injections” design. Robotic arm positioning allowed precise alignment with the planned trajectories. The abscesses were aspirated, and each cavity was thoroughly irrigated with gentamicin-saline until the effluent was clear. The needle was then withdrawn along the same trajectory to minimize the risk of seeding or infection.

**Fig. 3:**
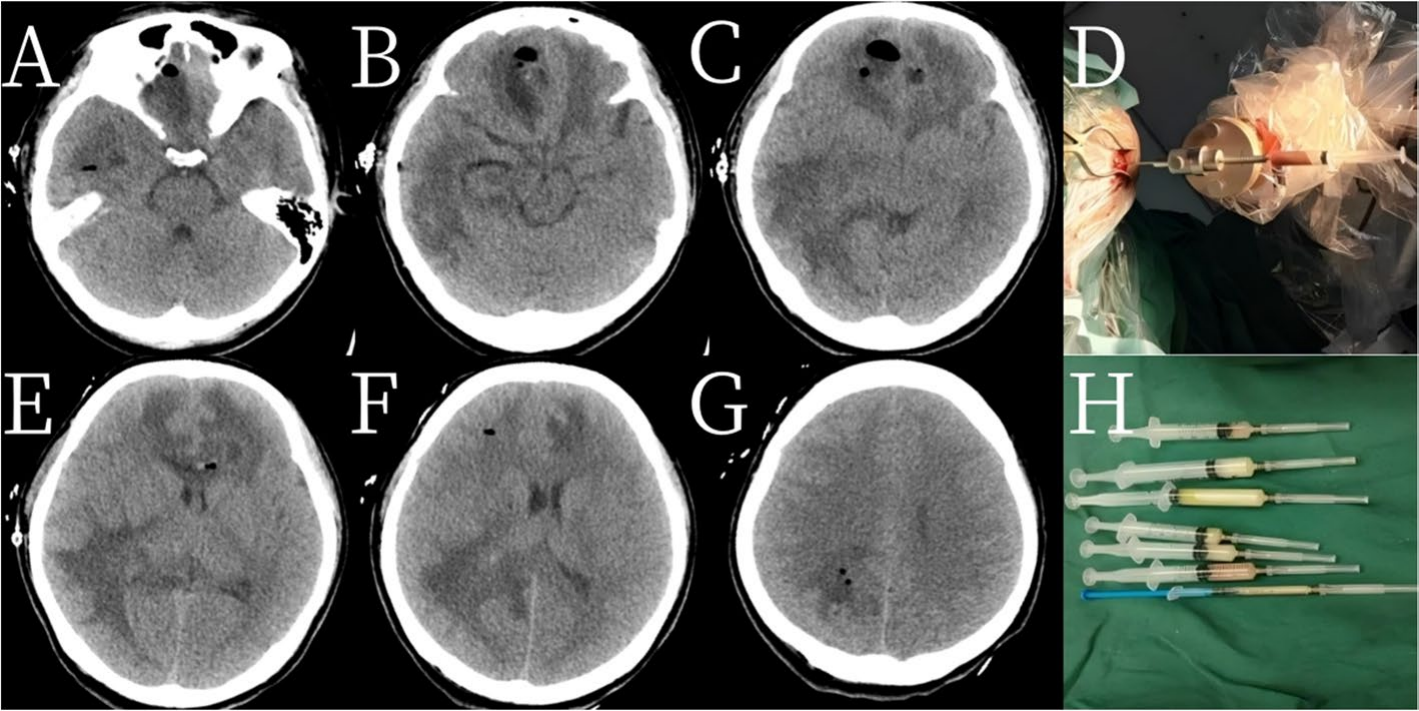
**A**–**C**, **E**–**G** Postoperative CT scans demonstrating successful aspiration of the brain abscesses with restoration of midline structures and no evidence of hemorrhage. **D** Intraoperative image of a stereotactic puncture. **H** Intraoperative aspiration of the purulent material

An immediate postoperative CT confirmed the restoration of midline structures without hemorrhage (Fig. 3).

**Fig. 4:**
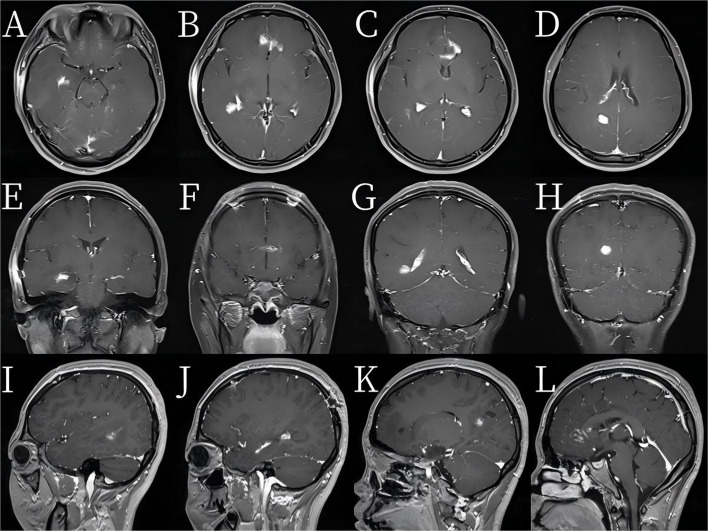
Complete resolution of the original abscesses is observed in the bilateral frontal lobes, genu of the corpus callosum, and right temporoparietal region, with near-complete disappearance of the abscess walls

**Fig. 5:**
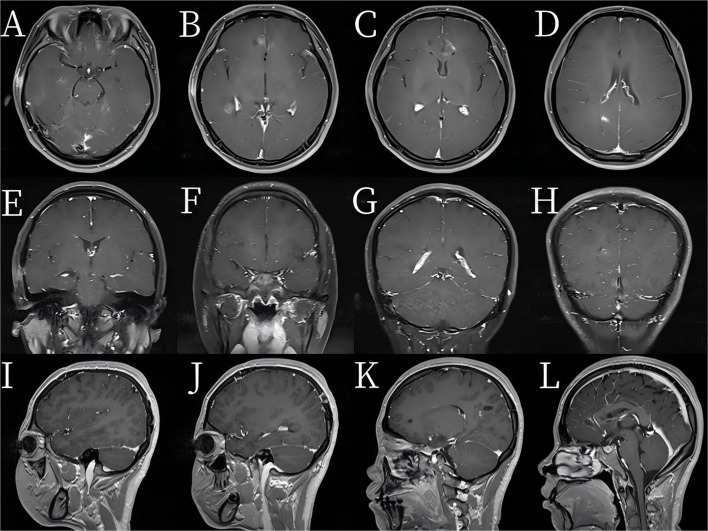
Compared with those in previous images, abnormal patchy signals in the bilateral frontal lobes, genu of the corpus callosum, and right temporoparietal region are markedly reduced

Empirical broad-spectrum antibiotics (meropenem + vancomycin) were started preoperatively in accordance with the institutional protocol for severe intracranial infections and adjusted after culture revealed *Streptococcus intermedius*, with susceptibility testing supporting narrowing of therapy. After 2 weeks of intravenous therapy, the regimen was transitioned to oral linezolid. Because the patient had significant perifocal edema and mass effects, we administered a short-course of low-dose dexamethasone (carefully monitored) to reduce edema and prevent secondary neurological deterioration. The patient’s fever and meningeal signs improved within 72 h, and consciousness recovered rapidly. One month after surgery, MRI showed that the abscess had completely disappeared and that the wall of the abscess had almost completely vanished (Fig. 4). The patient fully recovered, with a KPS score of 100 and no complications such as epilepsy. At the 3-month follow-up, MRI confirmed full recovery. During the 6-month and 1-year follow-up periods, the disease did not recur (Fig. 5).

## Discussion

If not promptly treated, brain abscess remains a severe intracranial infection associated with significant morbidity and mortality. Multifocal and deep-seated abscesses represent a particularly challenging clinical scenario because of their complex anatomical distribution, the presence of extensive cerebral edema, and the risk of rapid neurological deterioration.

Traditional surgical management of multifocal abscesses often involves drainage of the dominant lesion combined with antimicrobial therapy, while smaller lesions are treated conservatively or through staged procedures. Although this strategy reduces surgical invasiveness, it may delay the definitive treatment of remaining infectious foci and prolong the intracranial mass effect. Stereotactic aspiration has therefore become an important minimally invasive alternative, allowing accurate access to deep lesions while minimizing injury to surrounding brain tissue. In recent years, robot-assisted stereotactic systems have been increasingly introduced into neurosurgical practice [2]. Compared with conventional frame-based stereotactic techniques, robotic systems allow the automated positioning of surgical instruments according to preplanned trajectories with high reproducibility and submillimeter accuracy [3–5]. While frame-based stereotactic systems remain highly accurate, they often require repeated manual adjustments when multiple trajectories are needed. Frameless neuronavigation systems provide flexibility but may be influenced by surgeon-dependent positioning and registration variability.

The literature addressing the stereotactic management of multifocal brain abscess remains limited, and reports describing the robot-guided aspiration of multiple abscesses in a single procedure are particularly rare. As summarized in Table 1, the literature directly addressing the use of robot-assisted drainage for multifocal brain abscesses remains sparse. Earlier reports on multifocal abscesses have mainly relied on frame-based stereotactic aspiration, often targeting the dominant lesion or using staged procedures, whereas contemporary robotic reports suggest potential advantages in targeting reproducibility, workflow efficiency, and rapid multitarget access. The present case expands this limited body of evidence by demonstrating single-session aspiration of six abscess cavities through four robot-planned trajectories.

**Table 1:**
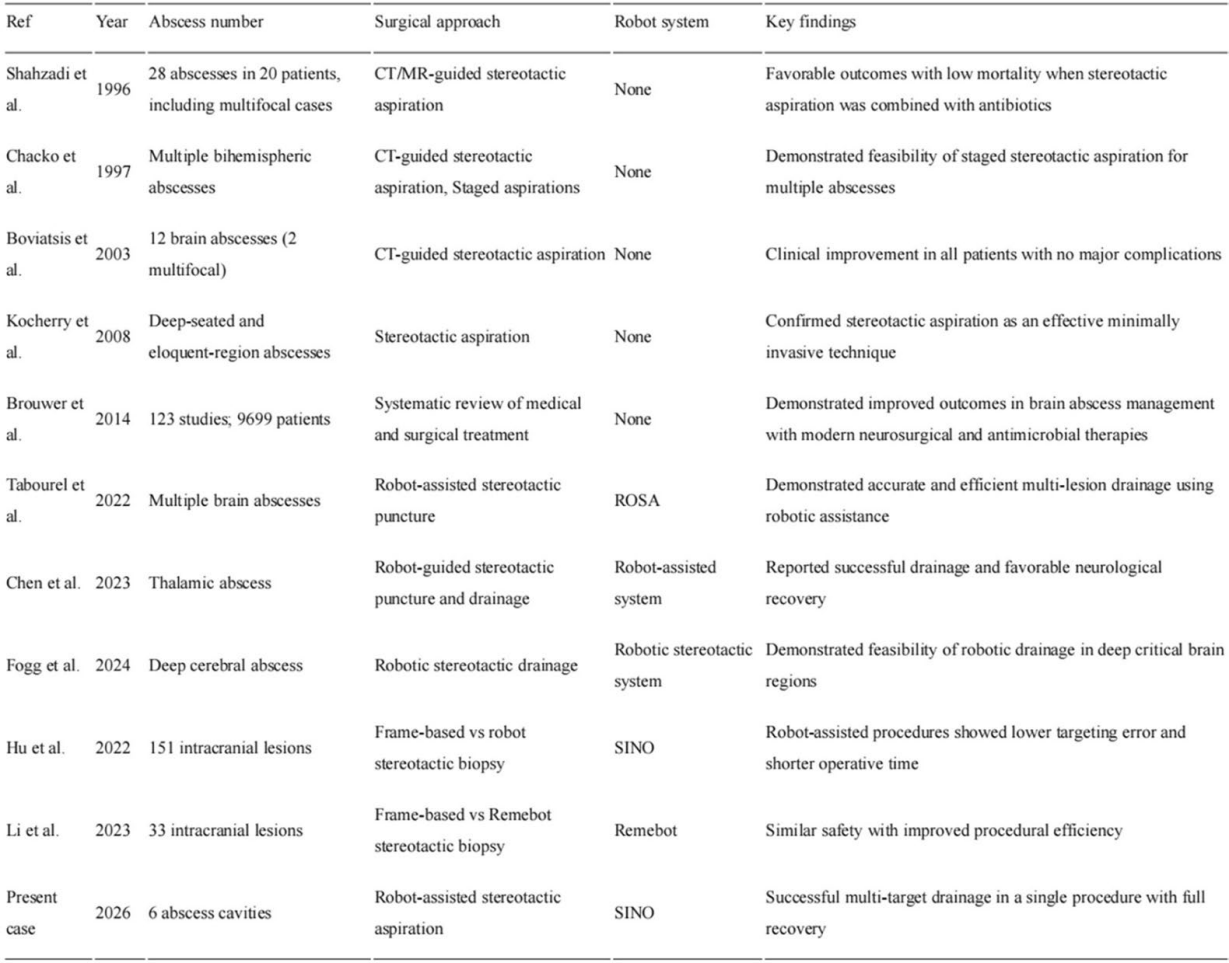
Representative studies [5–14]

Because direct literature on robot-assisted aspiration of multifocal brain abscesses remains limited, this table includes both disease-specific studies and methodologically relevant robotic stereotactic studies involving intracranial lesions

Preoperative MR–CT fusion allowed accurate visualization of major vessels and ventricular boundaries, enabling safe trajectory planning [15]. Sequential access to multiple lesions through shared trajectories minimized the number of cortical entry points and reduced surgical invasiveness. This approach allowed a rapid reduction in the intracranial mass effect while simultaneously obtaining material for microbiological diagnosis.

Potential risks associated with multitrajectory aspiration should also be considered. Multiple puncture paths may theoretically increase the risk of inflammatory reactions or abscess seeding along the puncture tracts. Careful trajectory planning and minimizing the number of cortical entry points are therefore critical. After the pus has been drained, each cavity should be thoroughly rinsed with gentamicin-saline, and the needle should be withdrawn along the same path to reduce the risk of infection or abscess seeding.

An important therapeutic consideration in this case was the judicious short-course use of low-dose dexamethasone to control life-threatening cerebral edema while avoiding prolonged immunosuppression that might impair infection clearance. Although extended corticosteroid therapy is generally discouraged in intracranial infections because of potential adverse effects, carefully selected short-course administration in the setting of effective drainage and adequate antimicrobial therapy may reduce mass effect and stabilize patients at risk of herniation [16, 17]. Such a pragmatic balance has been increasingly acknowledged in recent clinical series.

Another key aspect of management is the integration of precise surgical drainage with appropriate antimicrobial therapy. Empirical broad-spectrum antibiotics were initiated early and subsequently adjusted according to microbiological results, which identified *Streptococcus intermedius*. The combination of early drainage and targeted antimicrobial therapy is widely considered essential for successful brain abscess treatment [16].

Despite its advantages, robotic stereotactic surgery has several limitations. Registration errors during image fusion may lead to targeting inaccuracies, particularly in the presence of intraoperative brain shift. In addition, robotic systems require specialized equipment, additional training, and increased costs, which may limit their widespread adoption.

## Conclusion

This case demonstrates that robot-assisted stereotactic aspiration may represent a feasible and potentially effective strategy for managing multifocal deep-seated brain abscesses. By enabling precise multitarget trajectory planning and minimally invasive drainage within a single procedure, robotic assistance may facilitate rapid reduction of the intracranial mass effect while minimizing surgical trauma. Larger clinical studies are needed to further evaluate the role of robotic stereotactic techniques in the management of complex intracranial infections.

## Data Availability

All data generated or analyzed during this case report are included within the published article. No additional datasets or materials were generated that require separate deposition or access.
